# Characterization of *Undaria pinnatifida* Root Enzymatic Extracts Using Crude Enzyme from *Shewanella oneidensis* PKA 1008 and Its Anti-Inflammatory Effect

**DOI:** 10.4014/jmb.1908.08019

**Published:** 2019-12-02

**Authors:** Xiaotong Xu, So-Mi Jeong, Ji-Eun Lee, Woo-Sin Kang, Si-Hyeong Ryu, Kwangwook Kim, Eui-Hong Byun, Young-Je Cho, Dong-Hyun Ahn

**Affiliations:** 1 Department of Food Science and Technology and Institute of Food Science, Pukyong National University, Busan 4853, Republic of Korea; 2 Institute of Fisheries Sciences, Pukyong National University, Busan 46041, Republic of Korea; 3 Department of Food Science and Technology, Kongju National University, Chungnam 249, Republic of Korea; 4 School of Food Science and Biotechnology, Kyungpook National University, Daegu 1566, Republic of Korea

**Keywords:** *Shewanella oneidensis* PKA1008, *Undaria pinnatifida* roots, alginate degrading ability, anti-inflammatory

## Abstract

This study investigated the characterization and functionality of *Undaria pinnatifida* root (UPT) extracts, degraded using a crude enzyme from *Shewanella oneidensis* PKA1008. To obtain the optimum degrading conditions, the UPT was mixed with alginate degrading enzymes from *S. oneidensis* PKA 1008 and was incubated at 30°C for 0, 3, 6, 12, 24, and 48 h. The alginate degrading ability of these enzymes was then evaluated by measuring the reducing sugar, viscosity, pH and chromaticity. Enzymatic extract at 24 h revealed the highest alginate degrading ability and the lowest pH value. As the incubation time increased, the lightness (L *) also decreased and was measured at its lowest value, 39.84, at 12 hours. The redness and yellowness increased gradually to 10.27 at 6 h and to 63.95 at 3 h, respectively. Moreover, the alginate oligosaccharides exhibited significant anti-inflammatory activity. These results indicate that a crude enzyme from *S. oneidensis* PKA 1008 can be used to enhance the polysaccharide degradation of UPT and the alginate oligosaccharides may also enhance the anti-inflammatory effect.

## Introduction

Recently, as living standards have improved, there has been an increasing demand for natural foods due to the preference for healthy food intake, appropriate diet, and diversification [1]. In addition, as the stress levels increase, diseases such as obesity, hypertension, arteriosclerosis, diabetes, and cardiovascular disorders are mostly observed in adults; hence, health supplements from natural sources seem to be more promising in prevention and treatment of such diseases compared to drugs or medical treatment. Therefore, seaweed raw materials are gaining immense attention as a source of dietary supplements and physiologically active substances, and the utilization rate of seaweeds is assumed to continuously increase further [2].

Alginate-derived oligosaccharides are reported to have various antioxidant [3], anti-inflammation [4], and anti-blood coagulation properties [5]; however, alginate has a long solubility time at room temperature, and its viscosity is enhanced with increasing concentration, which limits its industrial use [6]. Studies on the degradation of seaweed polysaccharides are in process, thus aiming to solve these issues. Oligosaccharidization methods of polysaccharides are known to be hydrolysis, irradiation, chemical degradation and enzymatic degradation etc [7]. Since alginate is stabilized in acidic or alkaline conditions, it is difficult to decompose and it is difficult to set the decomposition conditions upon thermal decomposition. In addition, the irradiation with gamma rays is expensive and is not preferred by the consumers. Therefore, several studies were conducted on enzymes that specifically decompose alginate.

*Undaria pinnatifida* belongs to the brown algae group and is distributed mainly in Far East Asia including Korea, China, and Japan; these algae are rich in minerals, vitamins, and possess various useful nutrients [8, 9]. The alginate content in *U. pinnatifida* was reported to be the highest (35%) in *U. pinnatifida* root and sporophyll [10]; however, *U. pinnatifida* roots cannot be used as processed and remnant by-products, and most of them are disposed, thus leading to environmental pollution. Although these roots are known to have anti-inflammatory effects [11], no studies have reported high value-added and anti-inflammatory properties via degradation by microbial-based enzyme treatment. Therefore, in this study, *U. pinnatifida* roots was decomposed into low molecular weight oligosaccharides by the crude enzyme from *S. oneidensis* PKA 1008 in order to utilize *U. pinnatifida* roots by-products. Furthermore, we investigated the properties of the degraded alginate oligosaccharide and its anti-inflammatory effect as well as we tried to find out the possibility in being used as a natural anti-inflammatory agent.

## Materials and Methods

### Seaweed

UPT samples were supplied by Seokha Corp., Busan, Korea. It was removed with the tap water such as sand and salt attached to the surface of the sample. After natural drying for 1 day, it was lyophilized, then powdered and packed in vacuum and stored at -20°C.

### Strain

The strains used in this study were collected from Busan, Song-Jeong, Korea and were isolated from algae *Ulva pertusa*, which is undergoing degradation [6]. This was analyzed and identified using 16S rRNA sequence analysis.

### Preparation of Crude Enzyme from *S. oneidensis* PKA 1008 Strain

*S. oneidensis* PKA 1008 strain was cultured at 30°C for 24 h in marine broth (Difco Laboratories, USA), supplemented with 2%NaCl at pH 9, and was then centrifuged at 10,000 ×*g* for 30 min at 4°C. The supernatant was used as crude enzyme solution, and its activity was 490 U/ml.

### Preparation of UPT Enzymatic Extracts

UPT enzymatic extracts were prepared by method of Sunwoo *et al*. [6]. UPT samples were prepared at a concentration of 80 mg/ml using 10 mM phosphate buffer (pH 9). The optimum pH of the crude enzyme solution from *S. oneidensis* PKA 1008 was adjusted to pH 9 with 1 N NaOH. Next, 80 mg/ml UPT sample was mixed with the crude enzyme solution at a ratio of 1:1 (v/v). Simultaneously, sodium azide (0.02%) was added to prevent microbial contamination. The UPT enzymatic degradations were performed at 30°C for 0, 3, 6, 12, 24, and 48 h.

### Measurement of pH and Chromaticity

The pH change in the UPT enzymatic extract was measured with a pH meter (TOA, Japan).

The chromaticity of the UPT enzymatic extract was measured by using a colorimeter (Color Technosystem Co., Japan) after collecting the degradation product in a liquid sample cell. Each chromaticity is represented by the values of brightness (L *), redness (a *), and yellowness (b *), and the standard L*, a*, and b* values were 98.98, 0.21, and −0.28, respectively. The measurements were repeated minimum five times and the average values were obtained.

### Measurement of Reducing Sugar and Viscosity

To determine the changes in the content of reducing sugars in UPT enzymatic extracts, we used the Somogyi–Nelson method [12]. About 0.5 ml of sample and 0.5 ml of Somogyi’s alkaline copper solution were placed in a test tube and heated in a water bath for 20 min to form Cu_2_O; next, 1 ml of molybdic acid solution (MoO_3_·H_2_O·H_2_SO_4_) was added to develop color. Thereafter, the absorbance was measured at 520 nm using a spectrophotometer (Thermo Scientific, USA), and the reducing sugar was quantified with a calibration curve prepared with glucose as the standard.

The viscosity was measured at 25°C by a Stevens and Levin method [13] using a viscometer and 40 cP spindle at 30 rpm.

### Degradation of UPT Enzymatic Extracts

In order to inactivate the UPT enzymatic extract solution according to its incubation time, it was heated in boiling water for 10 min, centrifuged at 10,000 ×*g* for 10 min at 4°C, and then the supernatant was lyophilized. The lyophilized sample was dissolved in distilled water at a concentration of 20 mg/ml. Thereafter, ethanol (99.5%) was added to the sample (ethanol: sample = 4.1:1 (v/v)) and the crude sugar was extracted for 12 h. The supernatant was removed via centrifugation at 6,200 ×*g* for 30 min at 4°C and was then dried at room temperature to remove the ethanol. Low molecular weight degradation products of UPT by crude enzymes from *S. oneidensis* PKA 1008 were identified via thin layer chromatography.

### Cell Culture

The murine macrophages RAW 264.7 cells were purchased from Korean Cell Line Bank (KCLB 40071). The cells were cultured at 37°C in the presence of 5% CO_2_ in Dulbecco’s modified Eagle’s medium (DMEM; Welgene, Korea), supplemented with 10% fetal bovine serum (FBS), 100 U/ml penicillin, and 100 μg/ml streptomycin.

### Measurement of Pro-Inflammatory Cytokines Production

The levels of proinflammatory cytokines (IL-6, IL-1β, TNF-α) were determined using an ELISA kit (R&D Systems, USA). Briefly, RAW 264.7 cells (5 × 10^4^ cells/ml) plated in 48-well plates were pre-incubated for 18 h, following which they were cultured with LPS (2 μg/ml) and 10 μg/ml UPT enzymatic extracts according to their reaction time (0, 3, 6, 12, 24, and 48 h) at 37°C for 24 h. The levels of IL-6, IL-1β, and TNF-α in the culture medium were measured via ELISA using anti-mouse of IL-6, IL-1β, and TNF-α antibodies as well as biotinylated secondary antibodies, as per the manufacturer’s instructions.

### Nitric Oxide Determination

RAW 264.7 cells were preincubated in 48-well plates (5 × 10^4^ cells/ml) for 18 h. Next, 2 μg/ml of LPS was treated, and the 10 μg/ml UPT enzymatic extracts according to their incubation time (0, 3, 6, 12, 24, and 48 h) were added and cultured in a 5%CO_2_ incubator (Sanyo, Japan) at 37°C for 24 h. Thereafter, the supernatant (100 μl) was mixed with 100 μl of Griess reagent (1%sulfanilamide: 0.1% naphthalene diamine dihydrochloride in 5%phosphoric acid = 1:1) and the culture was reincubated at room temperature for 10 min. The absorbance was measured at 540 nm using a microplate reader (Bio-Rad, USA) and the quantity of nitrite was calculated with standard curves of sodium nitrite (NaNO_2_).

### Statistical Analysis

Data is expressed as mean ± standard error of the mean (*n* = 3). Statistical evaluation was carried out using analysis of variance with SAS software (SAS Institute, USA), according to Duncan’s multiple range test (*p* < 0.05).

## Results and Discussion

### pH and Chromaticity of UPT Enzymatic Extracts

We measured the pH changes (0, 3, 6, 12, 24, and 48 h) depending on the incubation time of UPT enzymatic extracts. As presented in Table 1, the pH of UPT enzymatic extracts was significantly decreased with the increasing incubation time, and it was lowered to 7.00 for 24 h.

Alginate is known to be degraded into polymannuronate by the cleavage of the β-1,4-glycosidic bond by alginate lyase [14]. In a study by Haug *et al*. [15], it was confirmed that the pH of the hydrololysate obtained by hydrolysis of alginate was 2.85. In addition, a study on the characterization of *Sargassum coreanum* enzymatic extracts by the crude enzyme from *S. oneidensis* PKA 1008 revealed that the pH reduced with increasing degradation time [16].

Therefore, it is considered that as the incubation time increases, the degraded alginate is increased and the pH is gradually decreased. These results suggest that UPT alginate degradation can be predicted by adding the crude enzyme of *S. oneidensis* PKA 1008 to UPT. On the other hand, as the incubation time increases, the growth and contamination of the microorganisms may cause the decrease in pH; however, since sodium azide (0.02%) is added to prevent the contamination of the enzymatic extracts by the microorganism, therefore, the possibility that the decrease in pH is due to microbial contamination is considered to be insignificant.

The chromaticity of UPT enzymatic extracts was measured according to the incubation time (Table 2). As a result, with increasing incubation time, the value of redness (a*) increased, and it was measured as 10.27 at 6 h, which then gradually decreased to 8.27 for 24 h. In the case of yellowness (b*), the highest value was 63.95 at 3 h. The lightness (L *) decreased with increasing incubation time and gradually tended to darken. The lowest value at 12 h was measured as 39.84, and thereafter, it increased continuously to 44.96 at 48 h. This result is in accordance with the results of Bark *et al*. [3], which reported that lightness decreased and redness and yellowness were increased by the enzymatic reaction using a crude enzyme.

The color of seaweeds is mainly due to the chlorophyll and carotenoid pigments, and in particular, brown algae comprise enormous chlorophyll and fucosanthin pigments, and these pigments exhibit a unique color. The color of seaweeds was found to be affected by discoloration during processing and storage, thus affecting the color of the degradation products [17]. Therefore, it is considered that the color change of the degradation product after incubation is due to the pigment component derived from the seaweed root sample.

### Reducing Sugar and Viscosity of UPT Enzymatic Extracts

Changes in the reducing sugar and viscosity of UPT enzymatic extracts were measured according to the incubation time (Table 3). The reducing sugar content of UPT enzymatic extracts was significantly enhanced with the increasing incubation time, and was highest with 250.68 μg ml at 24 h. The initial viscosity of UPT enzymatic extracts was 1.31 cP, which was significantly decreased with the increasing incubation time and was found to be 1.13 cP at 48 h.

Kim *et al*. [18] reported that when the alginate was decomposed into low molecular weight by heating, the viscosity and average molecular weight significantly decreased with the increasing heating time. In addition, our results are in accordance with the results of the previously study [6], stating that when alginate was mixed with the crude enzyme from *S. oneidensis* PKA 1008 for 0–60 h, the reducing sugar content increased and the viscosity decreased with the increasing reaction time. Park *et al*. [16] reported that the alginate degrading crude enzyme degrades polysaccharides present in *Sargassum coreanum*, most efficiently at 48 h. In this study, when UPT was incubated with the crude enzyme from *S. oneidensis* PKA 1008, it presented the highest reducing sugar content and low viscosity at 24 h. These results suggest that the most efficient reaction time for crude enzyme from *S. oneidensis* PKA 1008 to degrade polysaccharide of UPT into low molecular weight is 24 h.

 It was also predicted that enormous polysaccharides and oligosaccharides of alginate were degraded at 24 h reaction. Moreover, it was predicted that the oligosaccharide and monosaccharide of alginate were efficiently degraded at 24 h.

### Degradation of UPT Enzymatic Extracts

**Fig. 1:**
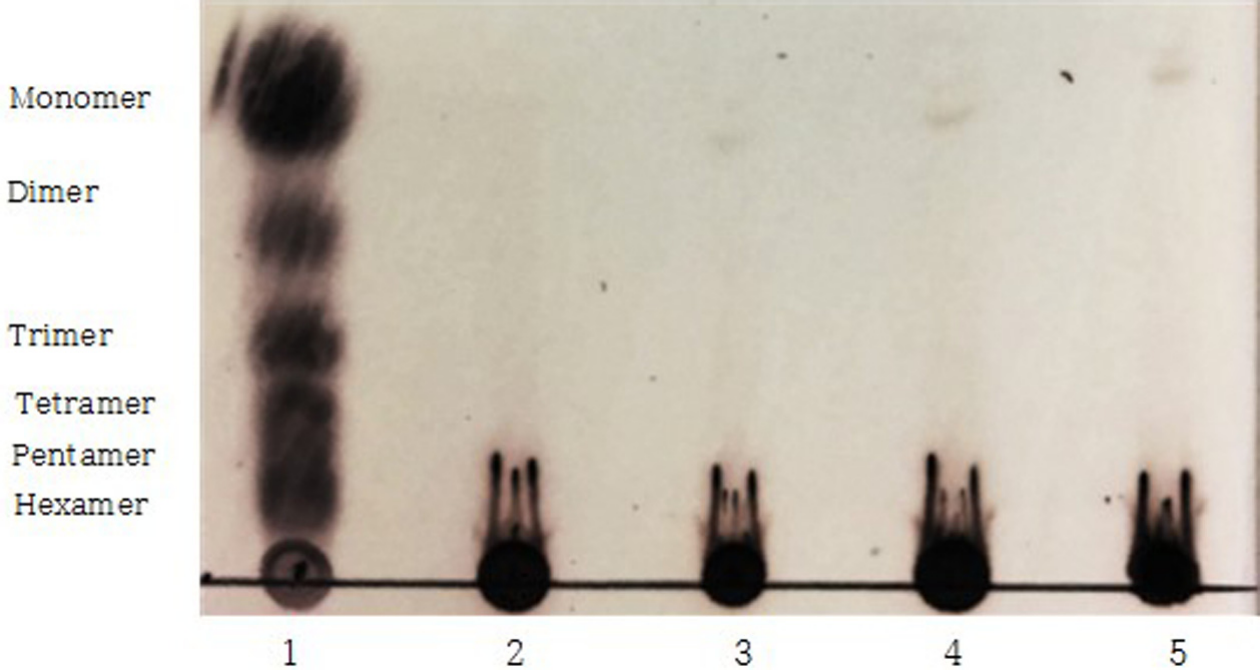
TLC analysis of alginate hydrolysate from *Undaria pinnatifida* roots by crude alginate degrading enzyme with various incubation time. Reaction time: lane 1, standard; lane 2, 0 h; lane 3, 3 h; lane 4, 24 h; lane 5, 48 h.

Thin layer chromatography (TLC) analysis was performed to confirm that UPT samples were degraded by the crude enzyme from *S. oneidensis* PKA 1008 treatment (Fig. 1). In the initial reaction, the degraded low molecules were not observed; however, after 3 h of reaction, one spot was observed at the monomer position and alginate was slowly degraded. It was observed that the spot concentration gradually increased until 48 h of the final reaction, and it was confirmed to degrade into monosaccharide at 48 h. This result was in accordance with the study of Kim *et al*.[19], which reported that the crude enzyme from *S. oneidensis* PKA 1008 degrades alginate to oligosaccharides and monosaccharides after 48 h of reaction. Therefore, in this study, when the crude enzyme from *S. oneidensis* PKA 1008 was treated with UPT, the polysaccharide was degraded into oligosaccharide and monosaccharide.

### Measurement of Pro-Inflammatory Cytokine Production

The macrophage RAW 264.7 cells were activated by the stimulation of an antigen such as LPS to regulate the immune response by promoting the secretion of cytokines such as IL-6, TNF-α, and IL-1β [19]. In this study, the inhibitory effect of UPT enzymatic extract on the inflammatory cytokines (IL-6, TNF-α, and IL-1β) was confirmed by the incubation time.

**Fig. 2:**
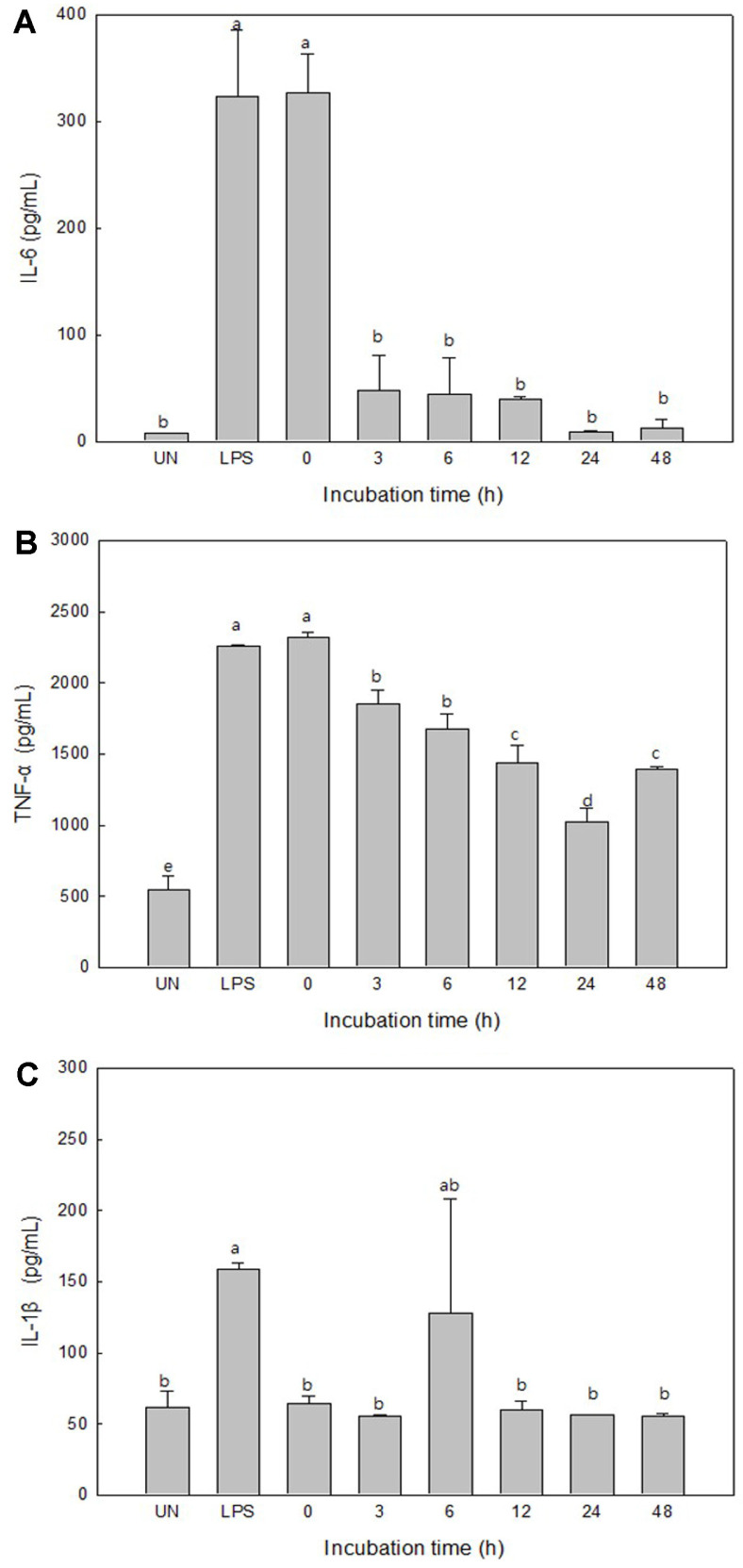
Inhibitory effect of enzymatic extracts of *Undaria pinnatifida* roots with incubation time on the production of IL- 6 (**A**), TNF-α (**B**), and IL-1β (**C**) in RAW 264.7 cells. ^a-d^Means with different superscripts are significantly different (*p* < 0.05).

As a result, it was confirmed that the amount of IL-6 secreted from the UPT enzymatic extract was significantly reduced in 3–48 h after culturing, compared with the LPS treatment group and the initial culture. The significant decrease in IL-6 secretion 3 h after the reaction was thought to be due to the degradation of the polysaccharides into monomers (Fig. 1). After, degradation then proceeded slowly, showing a reduction of about 97% of IL-6 secretion at 24 h (Fig. 2A). TNF-α also decreased with the increasing incubation time, and in particular, the UPT enzymatic extract treatment group presented a decrease of about 54%in TNF-α secretion compared to the LPS treatment group at 24 h of incubation (Fig. 2B). The amount of IL-1β secreted over time after the treatment with UPT enzymatic extract revealed reduction by about 62% after 12 h of incubation (Fig. 2C). These results are similar to those of the previous study [11] considering that the ethanol extract of *U. pinnatifida* roots reduces the inflammatory cytokines IL-6, TNF-α, and IL-1β secretion and has an inflammation-suppressing effect. Nevertheless, this study demonstrated that the treatment of *U. pinnatifida* roots with crude enzyme from *S. oneidensis* PKA 1008 can further improve the secretion inhibitory effect of inflammatory cytokines.

### Nitric Oxide Determination

Nitric oxide is a substance that mediates intercellular action and is produced by immune stimulation in macrophages or hepatocytes [20]. In this study, we investigated the effect of UPT enzyme extracts on NO production on LPS-induced RAW 264.7 cells for 0–48 h. As a result, the amount of NO secreted by LPS stimulation was 15.55 ± 1.30 μM, and the NO production was highly suppressed by the UPT enzymatic extracts treatment. In particular, the inhibition was significantly suppressed after 12 h and the amount of NO secretion was 4.72 ± 0.23 at 48 h, which was about 69% lower than that of the LPS treatment group.

Kang *et al*. [11] reported that the *U. pinnatifida* root ethanol extract treated group inhibited NO secretion by up to 34% compared to the LPS-treated control. On comparison, our results revealed that the UPT enzymatic extract was more effective in inhibiting NO secretion. These results suggest that the degradation of UPT by the crude enzyme from *S. oneidensis* PKA 1008 inhibits the secretion of NO from LPS-stimulated macrophages, and thus, the inflammation inhibition effect is greatly enhanced.

Therefore, the discarded *U. pinnatifida* roots can be used in several fields if they are treated with a crude enzyme from *S. oneidensis* PKA 1008 and are developed into a high value anti-inflammatory material.

## Figures and Tables

**Fig. 3:**
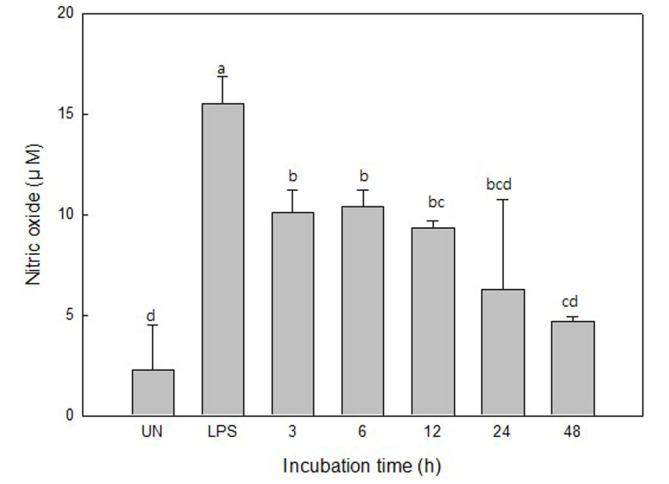
Inhibitory effect of enzymatic extracts of *Undaria pinnatifida* roots with incubation time on the production of NO in RAW 264.7 cells. ^a-d^Means with different superscripts are significantly different (*p* < 0.05).

**Table 1:** Changes in of pH enzymatic extracts of *Undaria pinnatifida* roots with incubation time.

Time (h)	pH
0	8.11 ± 0.00^a1)^
3	7.97 ± 0.01^b^
6	7.87 ± 0.01^c^
12	7.74 ± 0.01^d^
24	7.00 ± 0.01^e^
48	7.06 ± 0.01^f^

^1)^Means in the same column (a-f) bearing different superscript in samples are significantly different (*p* < 0.05).

**Table 2:** Changes in chromaticity of enzymatic extracts of *Undaria pinnatifida* roots with incubation time.

Time (h)	L*	a*	b*
0	59.28 ± 0.04^a1)^	5.17 ± 0.04^d^	52.50 ± 0.07^f^
3	43.68 ± 0.02^b^	9.98 ± 0.05^b^	63.95 ± 0.15^a^
6	41.35 ± 0.02^d^	10.27 ± 0.09^a^	62.16 ± 0.060^b^
12	39.48 ± 0.05^e^	9.92 ± 0.14^b^	60.60 ± 0.09^c^
24	43.03 ± 0.11^c^	8.27 ± 0.12^c^	57.90 ± 0.17^e^
48	44.96 ± 0.03^f^	8.29 ± 0.06^c^	59.15 ± 0.46^d^

^1)^Means in the same column (a-f) bearing different superscript in samples are significantly different (*p* < 0.05).

**Table 3:** Changes in reducing sugar assay and viscometry of enzymatic extracts of *Undaria pinnatifida* roots with incubation time.

Time (h)	Reducing sugar (μg/ml)	Viscosity (cP)
0	94.82 ± 0.83^c1)^	1.31 ± 0.02^a^
3	193.33 ± 1.25^b^	1.26 ± 0.04^b^
6	201.69 ± 2.10^b^	1.25 ± 0.03^b^
12	202.29 ± 5.45^b^	1.24 ± 0.02^b^
24	250.68 ± 1.78^a^	1.23 ± 0.02^b^
48	243.73 ± 1.73^a^	1.13 ± 0.04^c^

^1)^Means in the same column (a-c) bearing different superscript in samples are significantly different (*p* < 0.05).
